# *Gondwanamyia*, a new empidoid (Diptera) genus of uncertain placement

**DOI:** 10.3897/zookeys.621.10115

**Published:** 2016-10-03

**Authors:** Bradley J. Sinclair, Jeffrey M. Cumming, Scott E. Brooks, Adrian R. Plant, Toyohei Saigusa

**Affiliations:** 1 Canadian National Collection of Insects & Canadian Food Inspection Agency, OPL-Entomology, K.W. Neatby Bldg., C.E.F., 960 Carling Ave., Ottawa, ON, KIA OC6, CANADA; 2 Canadian National Collection of Insects, Agriculture and Agri-Food Canada, K.W. Neatby Bldg., C.E.F., 960 Carling Ave., Ottawa, ON, KIA OC6, CANADA; 3 Department of Biodiversity & Systematic Biology, National Museum of Wales, Cathays Park, Cardiff, CF10 3NP, UK; 4 7-1-402 Baikoen 2-chome, Chuo-ku, Fukuoka-shi 810-0035, Japan

**Keywords:** Empidoidea, Brachystomatidae, Trichopezinae, Empididae, Chile, New Zealand, new species, new genus

## Abstract

A new minute-size empidoid fly genus, *Gondwanamyia* **gen. n.** and two new species (*Gondwanamyia chilensis* Cumming & Saigusa, **sp. n.**, *Gondwanamyia zealandica* Sinclair & Brooks, **sp. n.**) are described, illustrated, and their distributions mapped. The family and subfamily assignments remain uncertain, but features of the female terminalia potentially suggest Trichopezinae (Brachystomatidae).

## Introduction

The higher classification of the exceedingly diverse Empidoidea, commonly referred to as dance flies and long-legged flies, consists of five well-defined families, namely Empididae, Hybotidae, Atelestidae, Brachystomatidae and Dolichopodidae s.l., plus three small unassigned genus-groups (Sinclair and Cumming 2006) that are sometimes given separate family status (i.e., Homalocnemiidae, Oreogetonidae) (Thompson 2009; Pape et al. 2011; Marshall 2012; Sinclair, in press), or probably soon will be (i.e., *Iteaphila* Zetterstedt group). There are still numerous new empidoid genera awaiting description that can all be placed confidently within these various groups. However, in this paper we report on a remarkable new empidoid genus that we cannot positively assign to either family or subfamily.

The genus contains two minute-size new species, one from southern Chile and the other from the North Island of New Zealand. Here we formally describe this new genus and its two included species. The potential phylogenetic placement of this curious Southern Hemisphere group within the Empidoidea, is also discussed.

## Material and methods

This study is based on material borrowed from or deposited in the following institutions: Canadian National Collection of Insects, Ottawa, Canada (CNC); Biosystematics Laboratory, Kyushu University, Fukuoka, Japan (KUMF); National Museum of Wales, Cardiff, UK (NMWC); New Zealand Arthropod Collection, Landcare, Auckland, New Zealand (NZAC). Label data for primary types are cited from the top downward, with the data from each label in quotation marks. Labels are cited in full, with original spelling, punctuation, and date, and label lines are delimited by a slash (/). The repository of each type is given in parentheses. Secondary type data are abridged and listed alphabetically.

Terms used for adult structures primarily follow Cumming and Wood (2009), except for the antenna and wing venation, where the terms of Stuckenberg (1999) and Saigusa (2006) are used, respectively. Male and female abdomens of certain specimens were macerated in hot 85% lactic acid and immersed in glycerin in order to examine terminalia.

## Taxonomy

### Gondwanamyia

Taxon classificationAnimaliaDipteraEmpididae

Sinclair, Cumming, Brooks, Plant & Saigusa gen. n.

http://zoobank.org/2AC98033-68E1-4C80-8C91-B07BB900C12E

#### Type-species.

*Gondwanamyia chilensis* Cumming & Saigusa, sp. n.

#### Diagnosis.

Body size minute, 1.1–1.4 mm. Arista-like stylus very long, longer than thorax, lacking basal article; males and females dichoptic, eye facets convex, appearing larger than in other empidoids; clypeus strongly convex; mouthparts with large epipharyngeal carina and paired epipharyngeal blades; wing with weakened fold near base, only two longitudinal veins fully developed (R_4+5_ and M_4_), R_4+5_ branched; abdomen with abdominal plaques; male terminalia symmetrical and unrotated, epandrium and hypandrium desclerotized and fused together, epandrial lobe and cercus projected anterodorsally, phallus tubular with or without an elongate apical filament, ejaculatory apodeme slender, rod-like; female terminalia sclerotized, sclerites beyond segment 6 mostly bare; tergite and sternite 8 anteroventrally articulated; female cercus sclerotized, projecting horizontally.

#### Description.

**Male.** *Head*: Dichoptic; eye facets convex; some short scattered ommatrichia. Antenna with scape reduced, strap-like with several setulae; pedicel globular, slightly shorter than postpedicel; arista-like stylus longer than thorax, lacking basal article. Ocellar triangle small, not raised, with stout ocellar setae inserted anteriorly, opposite anterior ocellus; several setulae posterior to ocellar triangle; vertical seta lateroclinate; 1 shorter seta between vertical seta and ocellar triangle; several postocular setae present. Mouthparts with swollen or inflated clypeus; epipharyngeal carina elongate and slender, extended well into clypeal region; epipharyngeal blades present at apex of carina, slender and pointed apically; labrum thickly sclerotized laterally; hypopharynx slender, stylet-like; lacinia apparently absent; palpus oval, flattened on inner face, with several long setulae; labellum pointed apically, narrow, without pseudotracheae, apical margin with series of peg-like sensilla.

*Thorax*: Chaetotaxy well developed and in distinct rows; acrostichals present or absent. Prosternum separated between fore coxae. Dorsal mesepimeral pocket present; metasternal furca tapered to narrow apex, lacking apical projections. Laterotergite bare. Fore leg simple, not raptorial; tibia without basal gland. Mid femur with row of stout posteroventral setae. Hind tibia with posteroapical comb.

*Wing*: Length: 1.1–1.5 mm. Cuneate and narrowed at base, slightly infuscate; anal lobe and alula not developed. Costa circumambient with basal costal seta and several subequal setae proximal to R_1_; slender, erect costal setae widely distributed to beyond R_4_; costal break distal to Sc, continuing posteriorly as weakened transverse fold across cell bm; costa with second break at R_1_; R_1_ very short, terminating in basal 0.25 of wing; R_2+3_ very short, nearly vertical, terminating at apex of R_1_; two longitudinal veins fully developed (R_4+5_ and M_4_); R_4+5_ branched, with R_4_ nearly perpendicular to R_5_; M_4_ nearly straight, slightly arched toward wing margin; cell dm absent; cell bm quadrate; cell br longer than cell bm; cell cua weakly open parallel to wing margin. Halter long, subequal in length to scutum; shaft with several basal setulae; knob tapered apically.

*Abdomen*: Abdominal tergites and sternites without modifications, sparsely setose; abdominal plaques present; tergite 8 slender, strap-like; sternite 8 expanded laterally to cradle terminalia, thinly sclerotized medioventrally; posterior marginal setae on sternite 8 distinct and well defined.

*Male terminalia*: Upright, symmetrical and unrotated; epandrium and hypandrium desclerotized and fused together; gonocoxal apodeme slender, rod-shaped; cercus well developed; phallus tubular with or without elongate apical filament, ejaculatory apodeme slender.

*Female terminalia*: Sclerotized, mostly bare; tergite 8 shallowly notched posteromedially with four posteromarginal setae; tergite and sternite 8 anteroventrally articulated; tergite ten subtriangular, not divided medially with one pair of posterolateral and one pair of apical setae, apical setae closely approximated; sternite ten triangular, pigmented laterally with pair of lateral setae; cercus thickly sclerotized, similar to sclerite 10, with apex bearing pair of long, stout setae; spermatheca thread-like, extending full length of abdomen.

#### Etymology.

Named after the southern Mesozoic continent of Gondwana, in reference to the probable age and distribution of this genus of flies on the southern continents of South America and Australasia (Figs 10, 11). All five authors are responsible for the new genus name. TS first identified this new genus, and both ARP and JMC + BJS independently studied separate series. SEB recently discovered the New Zealand species.

### Gondwanamyia chilensis

Taxon classificationAnimaliaDipteraEmpididae

Cumming & Saigusa sp. n.

http://zoobank.org/51E405D1-466A-4904-B4FB-914F911BE7B5

Figs 4–5 , 6 , 8 , 9 , 11

#### Type locality.

Chile: Los Lagos (Region X), Osorno Province, Puyehue National Park, Antillanca [ca. 40°46'S 72°12'W], 1300 m, *Nothofagus*, tree line.

#### Type-specimen.

**Holotype** male, pinned. Original label: “CHILE: Osorno, Puyehue/ N.P. Antillanca 1300m/ Feb. 11, 1988/ Nothofagus, tree line/ L. Masner, Chile Exp”; “HOLOTYPE/ *Gondwanamyia*/ *chilensis*/ Cumming & Saigusa” [red label] (CNC). **Paratypes**: **CHILE. Los Lagos (Region X)**. Chiloé Province: Chiloe Is., Ahoni Alto [ca. 42°46'S 73°34'W], 70 m, iv.1988, L. Masner (1 ♂, CNC). Llanquihue Province: N. Correntoso [ca. 41°26'S 72°35'W], N.E. Peurto Montt, vi-vii.1989 (2 ♂, 1 ♀, CNC). Osorno Province: Puyehue NP, Anticura [ca. 40°39'S 72°16'W], 250 m, *Nothofagus*, 12.ii.1988, L. Masner (15 ♂, 12 ♀, CNC); Puyehue NP, 1300 m, Antillanca [ca. 40°46'S 72°12'W], *Nothofagus*, tree-line, 11.ii.1988, L. Masner (18 ♂, 4 ♀, CNC). **Los Ríos (Region XIV)**. Valdivia Province: 4.1 km W Anticura, 270 m, 19–25.xii.1982, A. Newton & M. Thayer (5 ♂, 5 ♀, KUMF); 30 km W La Union, Las Trancas, *Nothofagus*, 500 m, 7–8, 25.ii.1988, L. Masner (2 ♂, 2 ♀, CNC). **Aysén del General Carlos Ibáñez del Campo (Region XI)**. Rio Gualas Sipi, Pto Gualas [ca. 46°31'S 73°42'W], 16.ii.1986, yellow pan traps in dense rainforest, N.A. Deans (1 ♀ in alc., NMWC).

#### Additional material examined.

**CHILE**. **Los Lagos (Region X)**. Osorno Province: Puyehue NP, Volcan Casablanca [ca. 40°43'S 71°57'W], 1370 m, nr. snowline, A. Newton & M. Thayer (1 ♂, CNC).

#### Recognition.

This species is distinguished from *Gondwanamyia zealandica* by the male mid femur and tibia with stout posteroventral setae, mostly membranous epandrium + hypandrium, and long phallic filament.

#### Description.

**Male.** *Head*: Ocellar setae stout, reclinate. Arista-like stylus slightly longer than 0.5 mm.

*Thorax*: Brown, darker than abdomen. Acrostichals absent; six dorsocentral setae; one postpronotal seta, two notopleural setae, upper longer and stouter; one presutural and two postsutural supra-alar setae; one postalar, elongate, subequal in length to apical scutellar seta; two pairs of scutellar setae, lateral pair slender and shorter than apical pair.

*Legs*: Mid femur with posteroventral row of 10–11 stout setae, longer than width of tibia (Fig. 5). Mid tibia with posteroventral row of apically-directed stout setae. Apex of hind tibia broadly expanded with posteroapical comb.

**Figures 1–5.**
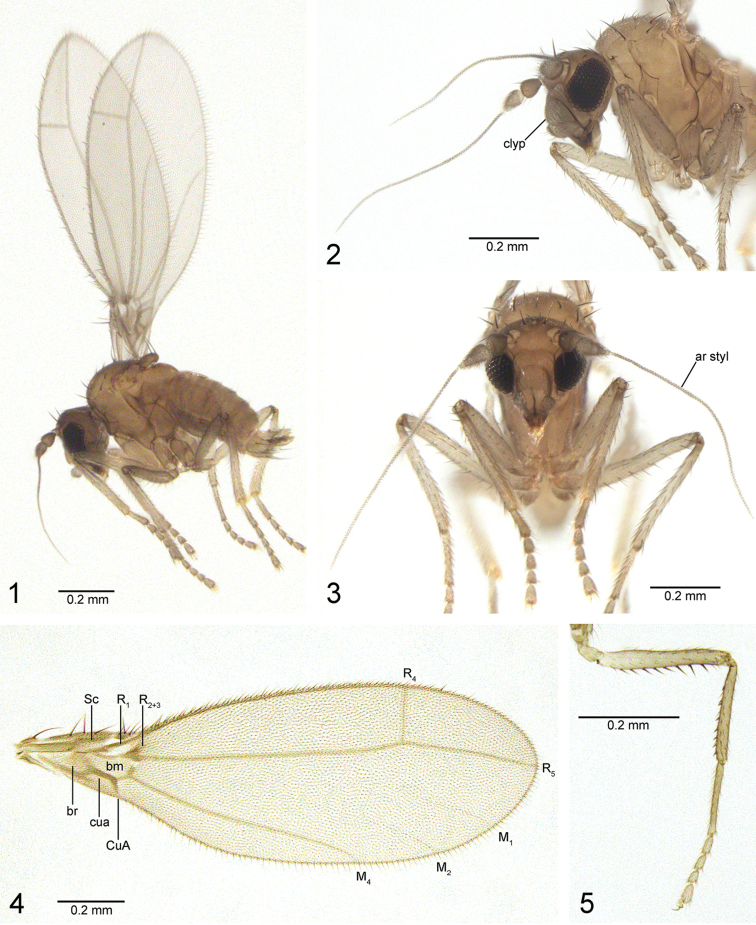
*Gondwanamyia*, habitus, wing and mid leg. **1–3** *Gondwanamyia zealandica* sp. n., holotype ♂ (in ethanol, prior to drying and mounting), habitus **4** *Gondwanamyia chilensis* sp. n., male wing **5** *Gondwanamyia chilensis*, male right mid leg, posterior view. Abbreviations: ar styl: arista-like stylus; bm: basal medial cell; br: basal radial cell; clyp: clypeus; CuA: anterior branch of cubital vein; cua: anterior cubital cell; M_1_, M_2_, M_4_: medial veins; R_1_, R_2+3_, R_4_, R_5_: radial veins; Sc: subcostal vein.

*Wing* (Fig. 4): Length 1.2–1.5 mm. Erect costal setae less conspicuous than in *Gondwanamyia zealandica*; faint subapical vein streaks present, representing veins M_1_ and M_2_.

**Figures 6–7.**
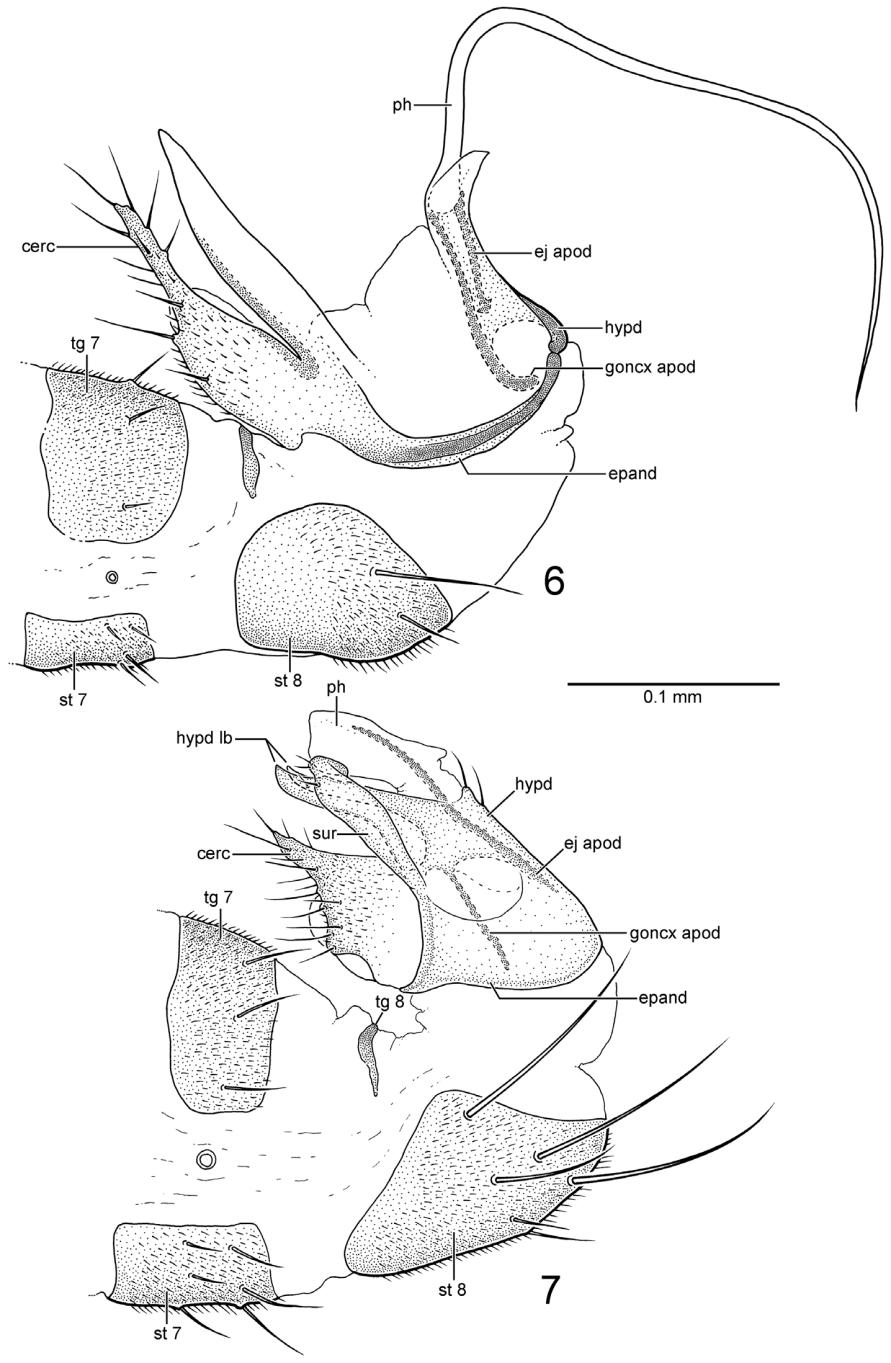
*Gondwanamyia*, male terminalia in lateral view. **6** *Gondwanamyia chilensis* sp. n. **7** *Gondwanamyia zealandica* sp. n. Abbreviations: cerc: cercus; ej apod: ejaculatory apodeme; epand: epandrium; goncx apod: gonocoxal apodeme; hypd: hypandrium; hypd lb: hypandrial lobe; ph: phallus; st: sternite; sur: surstylus; tg: tergite.

*Abdomen*: Two pairs of posterior setae on male sternite 8 short and slender. Male terminalia (Fig. 6): Cercus slender, apical half narrow and parallel-sided, bearing short, slender setae. Hypandrium + epandrium mostly membranous; slender epandrial lobe anterior, parallel with cercus, lacking setae, apex of lobes united medially; base of lobe and cercus continuous as dark, slender sclerite arching posteriorly to articulate with base of hypandrium. Hypandrium sheath-like, encircling base of phallus; gonocoxal apodeme slender, arched posteriorly, arising along interior of hypandrial sheath. Phallus with elongate apical filament, 3× longer than length of hypandrial sheath; ejaculatory apodeme rod-shaped, with expanded apex, articulated at base of phallus.

**Figures 8–9.**
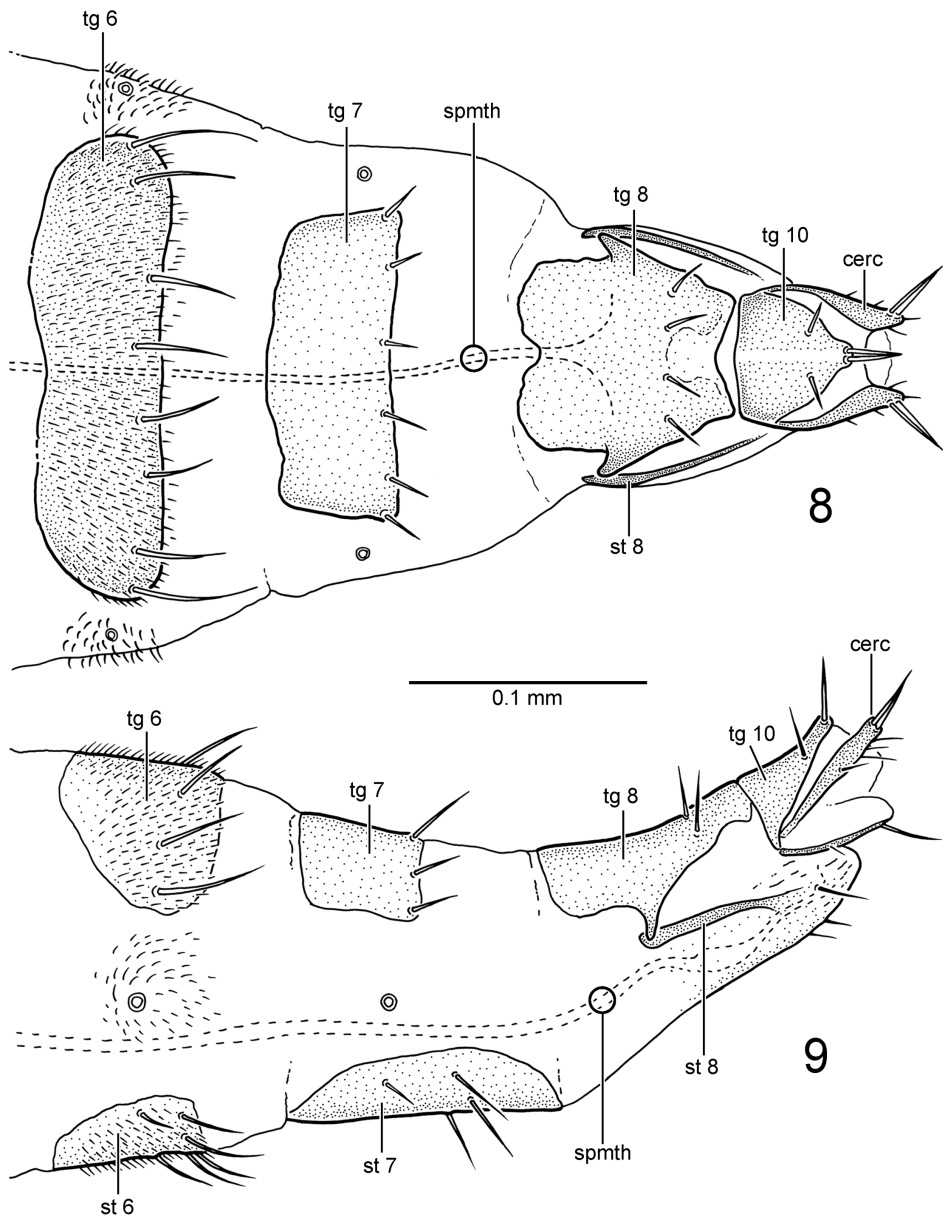
*Gondwanamyia chilensis* sp. n., female terminalia. **8** dorsal view **9** lateral view. Abbreviations: cerc: cercus; spmth: spermatheca; st: sternite; tg: tergite.

**Female.** Similar to male, except posteroventral row of stout setae on mid femur and tibia absent. See genus description for details of female terminalia (Figs 8, 9).

#### Etymology.

The species name refers to the country of the type locality.

#### Remarks.

This species is confined to southern Chile (Fig. 11) where it is found in mostly damp temperate *Nothofagus* habitats from lowlands to tree line. All specimens were collected with yellow pan traps, except the Newton & Thayer sample, which was collected with a flight-intercept trap.

**Figures 10–11.**
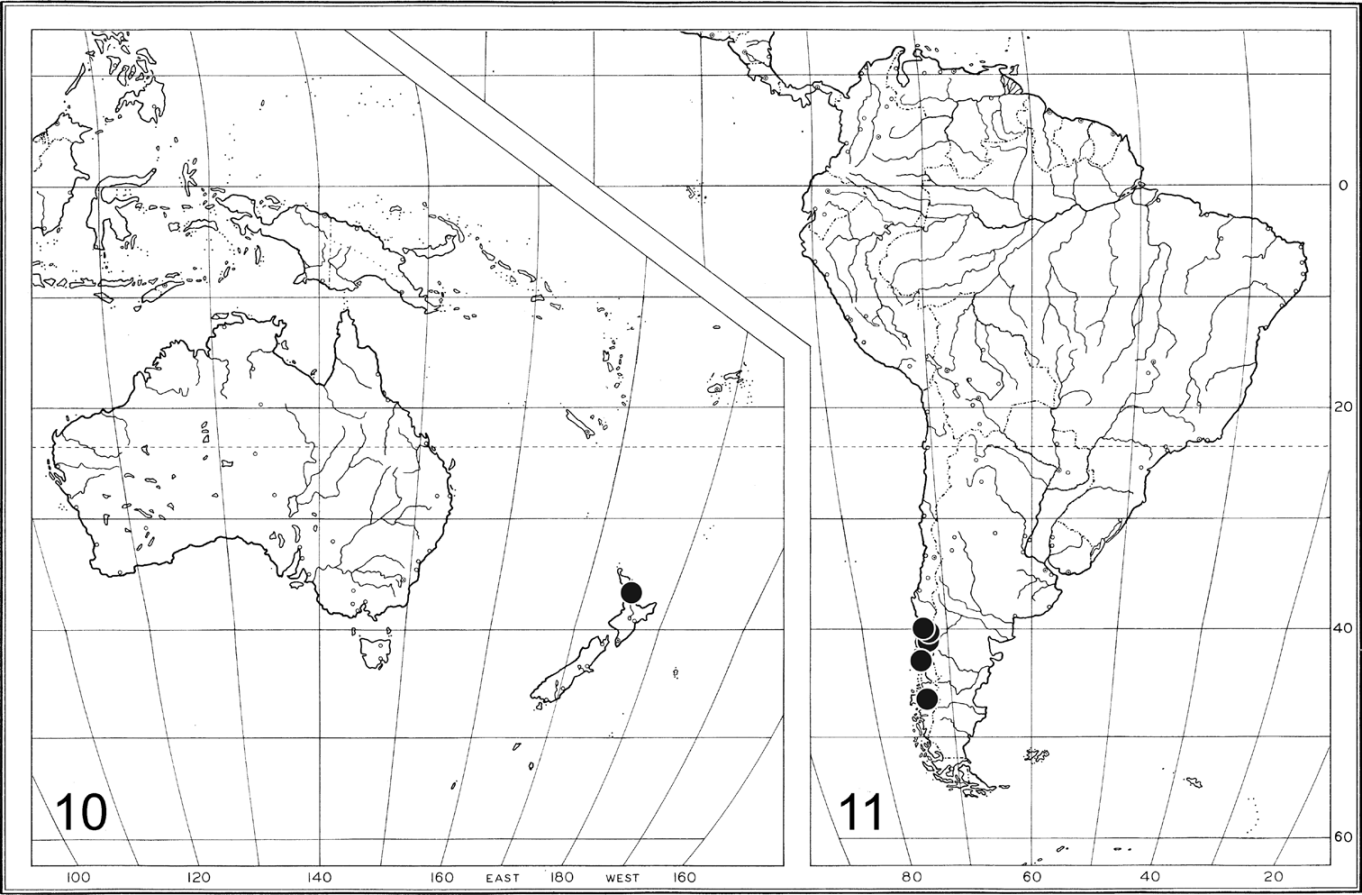
Distribution of *Gondwanamyia*. **10** Known distribution of *Gondwanamyia zealandica* sp. n. **11** Known distribution of *Gondwanamyia chilensis* sp. n.

### Gondwanamyia zealandica

Taxon classificationAnimaliaDipteraEmpididae

Sinclair & Brooks sp. n.

http://zoobank.org/A08B2505-A2DD-49B8-8DEE-D2EFE269C26C

Figs 1–3 , 7 , 10

#### Type locality.

New Zealand: Auckland: Waitakere Range, Matuku Reserve, 25 m, 36.867°S, 174.476°E.

#### Type-specimen.

**Holotype** male, pinned. Original label: “NEW ZEALAND: AK:/ Waitakere Range/ Matuku Reserve, 25 m/ 36.867°S, 174.476°E/ 2–5.iv.2010; L. Masner/ YPT [yellow pan trap] #23”; “HOLOTYPE/ Gondwanamyia/ zealandica/ Sinclair & Brooks” (NZAC).

#### Recognition.

This species is distinguished from *Gondwanamyia chilensis* by the less stout posteroventral row of setae on the mid femur and tibia, presence of acrostichal setae, arista-like stylus slightly shorter, posterior setae on male sternite 8 longer and stouter, and phallus without an apical filament.

#### Description.

**Male.** *Head*: Ocellar setae stout, reclinate. Arista-like stylus approximately 0.5 mm.

*Thorax*: Light brown, concolourous with abdomen. Acrostichal setulae present, at least three, uniserial; five dorsocentral setae, anterior seta stouter and laterally offset; one postpronotal seta, two strong, subequal notopleural setae; one presutural and three postsutural supra-alar setae; one postalar; two pairs of scutellar setae, lateral pair slender and shorter than apical pair.

*Legs*: Mid femur with posteroventral row of pale setae, longer than width of tibia. Mid tibia without posteroventral row of stout setae. Apex of hind tibia slightly expanded with posteroapical comb.

*Wing*: Length 1.1 mm. Erect costal setae more conspicuous than in *Gondwanamyia chilensis*; subapical vein streaks present, representing veins M_1_ and M_2_.

*Abdomen*: Four pairs of posterior setae on male sternite 8 long and stout. Male terminalia: Cercus broadly expanded at base, tapered to narrow apex; bearing fine setae. Hypandrium + epandrium with broad ventral connection; slender surstylus with pair of preapical setae; apex arched medially. Hypandrium sheath-like, with two pairs of tapered and apically pointed anterior hypandrial lobes; gonocoxal apodeme very slender, straight, arising on interior of hypandrial sheath. Phallus with hood-like apex, without apical filament; ejaculatory apodeme rod-shaped, very long and slender, running along posterior margin of hypandrium.

**Female.** Unknown.

#### Etymology.

The species name refers to the country of the type locality.

#### Remarks.

This species is known only from the type-locality, on the North Island of New Zealand (Fig. 10). The New Zealand habitat is apparently very different than that for *Gondwanamyia chilensis*, but collection of additional specimens and observations on the precise micro-habitat of this species in the Matuku Reserve are required.

## Discussion

Members of *Gondwanamyia* are among the smallest empidoid flies known. Based on the structure of their mouthparts, with free epipharyngeal blades, it is probable that they are predators on tiny arthropods, but nothing is currently known of their habits. Collection dates from both Chile (Dec., Feb., April, June, July) and New Zealand (April) indicate that this is primarily a late flying autumnal genus, apart from the December record. The weather in Chile and New Zealand during these months can be cool to cold. Most specimens were collected using yellow pan traps on the ground, indicating that members of this genus possibly fly low in the forest understory.

The small size of *Gondwanamyia* has presumably led to reductions in certain features such as wing venation. This has undoubtedly resulted in a loss of informative synapomorphies resulting in some confusion with homologies, creating uncertainty with the family and subfamily assignment of the genus. For example, the homologies of the male terminalia of both species are not completely resolved. The epandrium and hypandrium of *Gondwanamyia zealandica* appear fused together (Fig. 7), as presumably they are in *Gondwanamyia chilensis*, although with more desclerotization (Fig. 6). In addition, the hypandrial lobe in *Gondwanamyia zealandica* has a lateral and medial component that could include the postgonite, whereas the similarly positioned large undivided lobe in *Gondwanamyia chilensis* may also incorporate the surstylus.

*Gondwanamyia* obviously does not belong to the Hybotidae or Dolichopodidae s.l., based on presence of a branched R_4+5_ wing vein, unrotated male terminalia, and absence of a palpifer and fore tibial gland. It cannot be placed in the Atelestidae based on a branched R_4+5_ wing vein, setose scape, elongate hypandrium, and presence of female tergite 10. The genus is also clearly not assignable to either the *Homalocnemis* Philippi, *Oreogeton* Schiner, or *Iteaphila* genus (or family) groups.

*Gondwanamyia* might represent a new lineage of Empididae, although no convincing synapomorphies appear to align it with any included member of that family. The male terminalia of the new genus show some similarities to most Nearctic species of *Chelipoda* Macquart (Empididae: Hemerodromiinae) (MacDonald 1993, fig. 12), particularly in the fusion of the epandrium and hypandrium. However, no other features of the terminalia are shared between these two groups and not all species of *Chelipoda* exhibit the epandrial-hypandrial fusion (Plant 2007). Although wing venation reduction is also common among certain genera of Hemerodromiinae (Plant 1993, 2005), the pattern of reduction in both the Hemerodromiinae and in *Gondwanamyia* does not appear to be homologous. Males of the monotypic New Zealand hemerodromiine genus *Monodromia* Collin, also have a wing with a similar weakened fold near the base. The function of this weakening (present in both sexes in *Gondwanamyia*) is unknown in both genera.

The form of the female terminalia of *Gondwanamyia* (only known for *Gondwanamyia chilensis*) suggests inclusion in the Brachystomatidae based on the anterior articulation of tergite and sternite 8, the more thickly sclerotized terminal segments with absence of micro-setae, and well developed setae at the apex of the cerci and tergite 10 (Sinclair and Cumming 2006). If the genus does belong in this family, it is not assignable to the Ceratomerinae due to the absence of a conus on the antennal pedicel, or to the Brachystomatinae due to its shortened cell cua, absence of a lacinia and straight spermatheca (Sinclair and Cumming 2006). This implies that *Gondwanamyia* by default, probably belongs in the Trichopezinae. This subfamily is a very heterogenous group and many genera (including *Gondwanamyia*) lack the internal median apodeme that projects anteriorly from female tergite 8, which originally formed the basis for recognizing this lineage (Sinclair 1995). Further study and analysis of the Trichopezinae is required to determine more conclusively whether *Gondwanamyia* should be assigned to this lineage.

## Supplementary Material

XML Treatment for Gondwanamyia

XML Treatment for Gondwanamyia chilensis

XML Treatment for Gondwanamyia zealandica
